# Mutant superoxide dismutase aggregates from human spinal cord transmit amyotrophic lateral sclerosis

**DOI:** 10.1007/s00401-018-1915-y

**Published:** 2018-10-03

**Authors:** Elaheh Ekhtiari Bidhendi, Johan Bergh, Per Zetterström, Karin Forsberg, Bente Pakkenberg, Peter M. Andersen, Stefan L. Marklund, Thomas Brännström

**Affiliations:** 1 Department of Medical Biosciences, Umeå University, 90186 Umeå, Sweden; 2 Research Laboratory for Stereology and Neuroscience, Department of Neurology, Faculty of Health, Bispebjerg-Frederiksberg Hospital Copenhagen, and Institute of Clinical Medicine, University of Copenhagen, Copenhagen, Denmark; 3 Department of Pharmacology and Clinical Neuroscience, Umeå University, 90186 Umeå, Sweden

**Keywords:** Superoxide dismutase, Prion-like, Aggregation, Propagation, Motor neuron disease

## Abstract

Motor neurons containing aggregates of superoxide dismutase 1 (SOD1) are hallmarks of amyotrophic lateral sclerosis (ALS) caused by mutations in the gene encoding SOD1. We have previously reported that two strains of mutant human (h) SOD1 aggregates (denoted A and B) can arise in *hSOD1*-transgenic models for ALS and that inoculation of such aggregates into the lumbar spinal cord of mice results in rostrally spreading, templated hSOD1 aggregation and premature fatal ALS-like disease. Here, we explored whether mutant hSOD1 aggregates with prion-like properties also exist in human ALS. Aggregate seeds were prepared from spinal cords from an ALS patient carrying the *hSOD1*^G127Gfs*7^ truncation mutation and from mice transgenic for the same mutation. To separate from mono-, di- or any oligomeric hSOD1 species, the seed preparation protocol included ultracentrifugation through a density cushion. The core structure of *hSOD1*^G127Gfs*7^ aggregates present in mice was strain A-like. Inoculation of the patient- or mouse-derived seeds into lumbar spinal cord of adult hSOD1-expressing mice induced strain A aggregation propagating along the neuraxis and premature fatal ALS-like disease (*p* < 0.0001). Inoculation of human or murine control seeds had no effect. The potencies of the ALS patient-derived seed preparations were high and disease was initiated in the transgenic mice by levels of hSOD1^G127Gfs*7^ aggregates much lower than those found in the motor system of patients carrying the mutation. The results suggest that prion-like growth and spread of hSOD1 aggregation could be the primary pathogenic mechanism, not only in *hSOD1* transgenic rodent models, but also in human ALS.

**Electronic supplementary material:**

The online version of this article (10.1007/s00401-018-1915-y) contains supplementary material, which is available to authorized users.

## Introduction

Amyotrophic lateral sclerosis (ALS) is characterized by adult-onset degeneration of the upper and lower motor neurons. The paresis begins focally and spreads to adjacent myotomes, suggesting an early initial event in one motor unit followed by contiguous spread of the disease process [9]. The result is progressive paresis and inevitably death when the respiratory muscles become paralyzed. Mutations in the gene encoding the ubiquitously expressed cytosolic enzyme superoxide dismutase 1 (SOD1) cause ALS [32], and are found in 1–9% of patients [1]. To date, over 200 mutations in *SOD1* have been found in patients with ALS [37] (http://alsod.iop.kcl.ac.uk/). Most of them are missense among the 153 amino acids (aa) in the subunits of the SOD1 homodimer, but 23 cause long C-terminal truncations which preclude native folding. This suggests that any common ALS-inducing SOD1 species must be misfolded.

Neuronal inclusions containing aggregated SOD1 are a hallmark of ALS, both in patients and in transgenic (Tg) animal models expressing mutant human SOD1 s (hSOD1) [24]. We recently reported that two structurally different strains of aggregates (A and B) can arise in spinal cords of mice expressing full-length human SOD1 (hSOD1) variants [6]. They were different from hSOD1 aggregates generated under a variety of conditions in vitro, showing that the conditions in the CNS shape the aggregation process. Inoculation of strain A or B hSOD1 aggregates into spinal cord of asymptomatic mice expressing a *hSOD1* transgene triggered spreading templated hSOD1 aggregations and early-onset fatal ALS-like disease [7]. Seeding effects of whole homogenates of spinal cords from end-stage Tg mice have also been reported [4, 5]. Moreover, homogenates from two patients carrying the* hSOD1*^*A4V*^ mutation have been found to induce aggregation of yellow fluorescent protein-fused hSOD1^G85R^ in spinal cord slices from Tg mice [3]. Finally, homogenates of spinal cords from ALS patients carrying several different hSOD1 mutations have been found to trigger increased aggregation of green fluorescent protein-fused hSOD1 mutants expressed in human embryonal kidney cells [31]. These findings suggest that a prion-like disease transmission could be the primary pathogenic mechanism of SOD1-induced ALS.

To cause ALS-like disease within the short lifespan of mice, the hSOD1 variants have to be expressed at rates ~ 20-fold higher than that of the endogenous murine SOD1 [17, 18, 21], and the relevance of the murine models for human ALS has been questioned [27, 30]. The primary goal of the present investigation was to determine whether hSOD1 aggregates with prion-like properties also are present in the spinal cord of humans with ALS.

Autopsy material from a patient carrying the p.G127Gfs*7 (alias “G127X”) *SOD1* truncation mutation was used for the study [2]. The choice was based on our previous finding of comparatively large amounts of aggregates in ventral horns from ALS patients carrying that mutation [19, 20]. Aggregates formed in spinal cords of hSOD1^G127X^ Tg ALS model mice were found to have a strain A-like core structure. Inoculation of both murine and human SOD1^G127X^ aggregates into spinal cords of mice expressing a *hSOD1* transgene caused spreading strain A aggregation and aggressive premature fatal motor neuron disease, demonstrating for the first time the presence of hSOD1 aggregates with prion-like properties in human ALS.

## Materials and methods

### Patient and control

The family carrying the *hSOD1*^*G127X*^ mutation has been described [2]. At the age of 71 years, the patient developed symptoms of muscle weakness beginning in the truncal muscles. Following a typical progressive disease course ending with tetraparalysis and general wasting of skeletal muscles, he died 2 years later. The patient displayed both upper and lower motor neuron signs but no atypical features, was not cognitively impaired and did not suffer from other relevant diseases. There was no other hereditary predisposition than ALS, in particular not for Creutzfeldt–Jakob disease. The control patient suffered from epilepsy and died of an acute myocardial infarction at the age of 73 years. With informed consent from both patients and the next of kin, tissue was saved and immediately frozen at − 80 °C. The post-mortem times were 26 and 29.5 h, respectively. The study was approved by the Research Ethics Committee at Umeå University as well as the Umeå Regional Ethical Review Board and adhered to the principles of the Declaration of Helsinki.

### Mice

Hemizygous Tg mice that express hSOD1^G85R^ (line 148) were used as recipients for the inoculations [8]. The lifespan of this mouse line is 397 ± 49 days (*n* = 101) in our laboratory. There are no differences between the sexes: the lifespans of the females were 398 ± 60 days (*n* = 31) and of the males 397 ± 44 days (*n* = 70). For preparation of the strain B seed, *hSOD1*^*D90A*^ Tg mice were used [23]. The *hSOD1*^G127X^ Tg mouse strain was generated in house, and was homozygous for the transgene insertion [20]. The mouse strains were backcrossed > 30 generations in C57BL/6 mice. The use and maintenance of the mice and the experimental protocol described in this article were approved by the Umeå Regional Animal Research Ethical Board.

### Preparation of hSOD1 aggregate seeds by centrifugation through density cushion

Strain A, strain B and control seeds were prepared from spinal cords from end-stage spinal cords of *hSOD1*^*G85R*^ and *hSOD1*^*D90A*^ Tg mice and from a 100-day-old non-transgenic C57BL/6 mouse, respectively. A seed was likewise prepared from two end-stage *hSOD1*^*G127X*^ Tg mice. Human-derived seeds were prepared from lumbar ventral horn specimen (lamina IX) dissected from the hSOD1^G127X^ patient and the control.

Briefly, the protocol involved homogenization in 25 volumes of PBS containing 1% NP40 and 1 M guanidinium chloride using an Ultraturrax and sonication [7]. After clearing by a 1000×*g* centrifugation, the homogenates were layered on top of a 4 cm high 13% iohexol cushion (*δ* = 1.074) and centrifuged at 175,000×*g* for 1 h. Under these conditions, proteinous components with molecular masses > ~ 5 × 10^6^ Da will be sedimented into the pellets [34]. The seeds will, therefore, not contain monomeric, dimeric or any oligomeric hSOD1 species. The pellets were suspended by sonication in a small volume of PBS and stored in aliquots at − 80 °C. The mouse hSOD1^G85R^ and hSOD1^G127X^ seeds contained both 3 ng/µl (mutant) hSOD1. The total protein contents of the mouse hSOD1^G85R^, hSOD1^G127X^ and control seeds were 207 ng/µl, 385 ng/µl and 220 ng/µl, respectively.

A second seed (II) was prepared from the same *hSOD1*^*G127X*^-carrying patient using a subsequent modified protocol. 0.5 M guanidinium chloride was used instead of 1 M, and following sonication, the homogenate was diluted with 3.33 volumes water with 1% NP40 to achieve physiological ionic strength. Thereafter, it was sonicated again, loaded on top of the 4 cm density cushion and subjected to ultracentrifugation. Characterization of the human hSOD1^G127X^ seeds I and II, and the human control seed (prepared by protocol I) is shown in Supplementary Fig. S2.

### Inoculation of the hSOD1 aggregate seeds into lumbar spinal cord and monitoring of mice

The protocol used was somewhat modified compared with the previous study [7]. The recipient 100-day-old asymptomatic *hSOD1*^*G85R*^ Tg mice were anesthetized with 5% isofluorane in an induction chamber. The mice were then fixed on a small animal stereotactic frame (Kopf Instruments) and anesthesia maintained with 1.5–2% isofluorane via a facemask. Also, 0.005 µg Rimadyl per gram weight was injected s.c. approximately 15 min before starting surgery. Two small bilateral, longitudinal cuts on the back close to the spine were then made, making pockets for attachment of a stabilizing spine clamp. To reach the spinal cord, a 3–4 mm transversal cut through the muscles was made. Inoculation was done between two vertebrae after the meninges were punctured using a fine needle. The depth of insertion was controlled using a digital display console with 10-µm resolution (model 940-B, Kopf). One µl of the seeds was inoculated into the lumbar ventral horn on the left side at the L2–L3 levels. A Syringe model 75 RN Neuros with a sharp custom syringe (33/78/4)S 30° (Hamilton) was used, and the injection velocity was 0.125 µl/min controlled by an infusion pump (Legato 130, KD Scientific). The syringe was then slowly pulled out, the fascia sutured, and the skin closed using Tissue Adhesive (3M Vetbond). The duration of the surgical procedures was about 25 min, and in total, the mice were under anesthesia for about 40 min. To compensate for dehydration and any blood loss, 250 µl physiological NaCl was injected subcutaneously. Buprenorphine was injected 2–3 times at 12-h intervals until the mice were pain free.

No obvious sphincter or other bladder disturbances were observed in any of the operated mice, and the sensory response in their hind legs was normal. The mice were examined at least every third day, and weighed once a week. In the hSOD1 aggregate seed-inoculated mice, the onset was uniformly splaying of a hind leg. The mice were considered terminally ill when the hind legs were fully or almost fully paralyzed. In cases with more prominent foreleg symptoms (some control-preparation and non-inoculated Tg mice), severe eye infection in combination with severe foreleg weakness was the criterion for end-stage disease.

### Excluded mice

Seven mice were excluded and not reported in “Results”, “Discussion”, figures or the Table 1. One mouse died during surgery and another 3 days thereafter. One mouse control seed-inoculated mouse was euthanized 59 days after inoculation because of infection, one because of a back wound after 93 days, and one was found dead in the cage after 153 days, probably because of infection. One mouse hSOD1^G127X^ seed-inoculated mouse was found dead of unknown cause after 58 days. Finally, a human hSOD1^G127X^-I seed-inoculated mouse died with swollen belly after 159 days. None of these mice showed any paretic symptoms.

**Table 1:** Characteristics of the motor neuron disease in inoculated mice

	Inoculated seeds									
	Human						Mouse			
	HSOD1^G127X−I^		hSOD1^G127X − II^		Control		hSOD1^G127X^		Control	
	*n*		*n*		*n*		*n*		*n*	
Age at inoculation (days)	10	103 ± 3^A,ns^	11	106 ± 4^A,^*	18	102 ± 2	11	107 ± 7^B,^*	10	98 ± 10
Time to symptom onset (days)	10	172 ± 50^A,^**	11	138 ± 47^A,^****	18	268 ± 38	11	62 ± 21^B,^****	10	238 ± 46
Duration of disease (days)	10	45 ± 40^A,ns^	11	58 ± 48^A,ns^	18	28 ± 27	11	24 ± 18^B,ns^	10	45 ± 47
Time to fatal disease (days)	10	216 ± 49^A,^***	11	196 ± 31^A,^****	18	296 ± 33	11	86 ± 14^B,^****	10	284 ± 54
Age at fatal disease stage (days)	10	319 ± 49^A,^***	11	302 ± 32^A,^****	18	398 ± 33	11	194 ± 15^B,^****	10	381 ± 49
hSOD1 aggregates (μg/g wet weight)	5	34 ± 17^A,ns^		n.a.	5	32 ± 11	5	17 ± 3^B,^**	5	32 ± 7
Loss of weights (%)	10	28 ± 9^A,ns^	11	25 ± 9^A,ns^	18	26 ± 8	11	24 ± 5^B,ns^	10	21 ± 12

The table shows data for mice predetermined for lifespan analysis. Data are shown as mean ± SD

*n.a.* not analyzed

Symbols denote significance of differences between hSOD1^G127X^ and control seeds: ns, *p* > 0.05; *p ≤ 0.05; **p ≤ 0.01; ***p ≤ 0.001; ****p ≤ 0.0001

^A^Comparison between the human seeds

^B^Comparison between the mouse seeds. Amounts of hSOD1 aggregates in end-stage mice were analyzed in pools of spinal cord and brain stem. For loss of weights, see also Supplementary Fig. S4

### Antibodies

Antibodies to peptides in hSOD1 (corresponding to aas 4–20, 24–39, 41–49, 43–57, 48–57, 57–72, 80–96, 94–102, 100–115, 115–121, 131–153, 132–140 and 144–153) that were coupled to keyhole limpet hemocyanin were raised in rabbits and affinity purified with immobilized peptides as described (Ra-Abs) [15, 20]. Antibodies raised to the C-terminal end in hSOD1^G127X^, aas 111–127GQRWK and 123–127 GQRWK, were prepared in the same way. A chicken antibody (Ch-Ab) against aas 131–153 was also raised and affinity purified. A monoclonal mouse antibody (Mo-Ab) against aas 131–153 was produced using standard hybridoma technique. For muscle immunohistochemistry, an antibody against slow myosin was used (clone NOQ 7,5,4D; Cat no M8421, Sigma). For motor neuron staining and counting, an antibody against choline acetyltransferase (ChAT) (abcam EPR 16590) was used.

### Tissue homogenization and binary epitope-mapping assay for hSOD1 aggregate content and structure

**Fig. 1:**
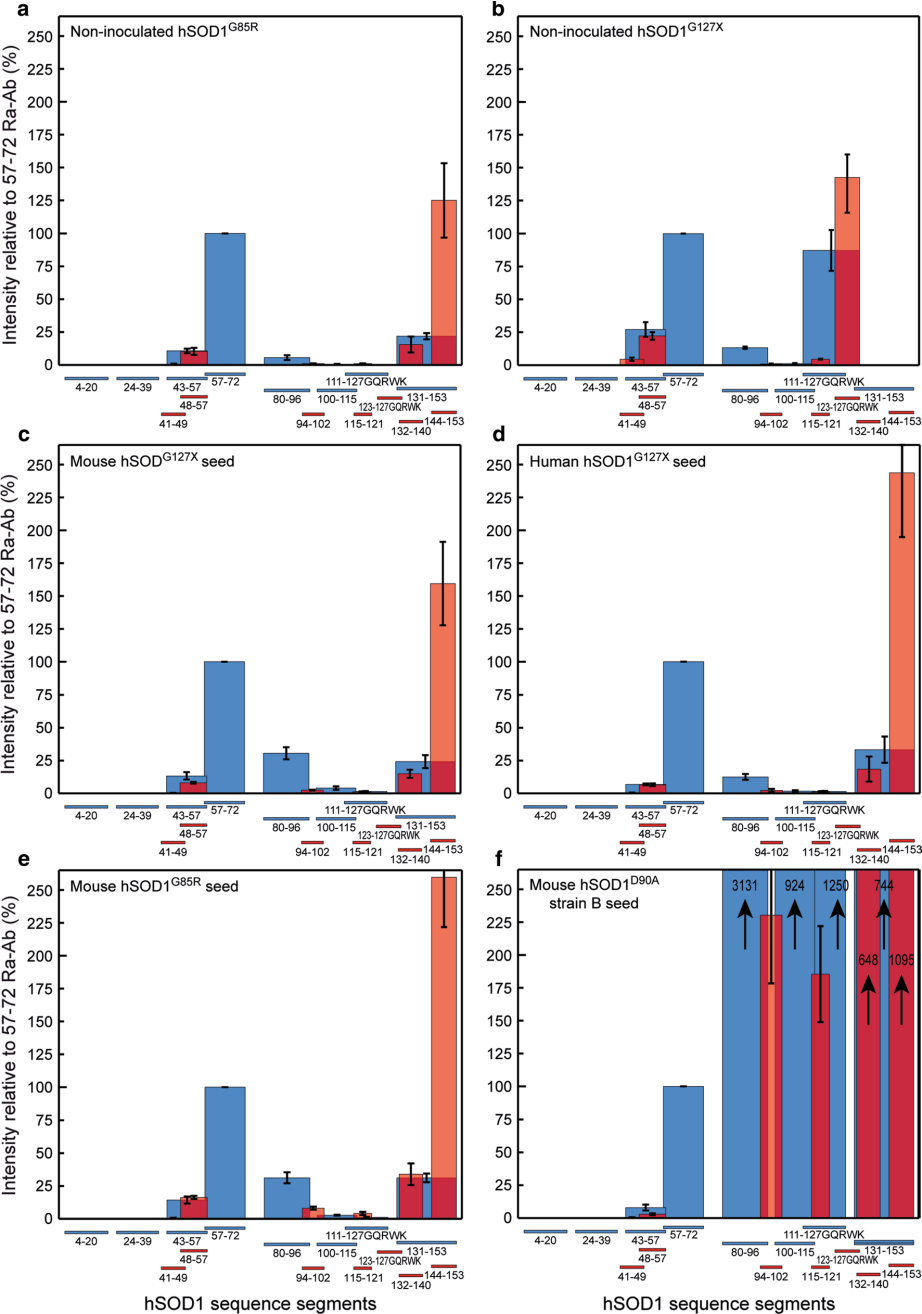
Binary epitope-mapping patterns of hSOD1 aggregates in spinal cords from end-stage non-inoculated and inoculated transgenic mice. Results for antibodies raised against longer SOD1 peptides used in the original protocol are labeled blue, and data for the additional antibodies raised against shorter peptides are labeled red. The aggregates were analyzed in 5 end-stage mice in all groups and the standard deviations and means are shown. To facilitate appreciation of patterns, all results were normalized against the staining intensity with the 57-72 Ra-Ab (= 100%). **a** and **b** Non-inoculated *hSOD1*^*G85R*^ and *hSOD1*^*G127X*^ Tg mice, respectively. **c**–**f** *hSOD1*^*G85R*^ Tg mice inoculated with indicated seeds

A detailed description of the background to the assay and the protocol is found in [6]. The current resolution-enhanced protocol is based on 15 rabbit antibodies raised against short peptides that cover over 90% of the 153 aa long hSOD1 subunits (Fig. 1). The reaction of the antibodies with aggregates captured on filter is determined. Since the configurational space of short peptides is very large, their randomly induced antibody-eliciting epitopes are unlikely to match defined ordered structures in proteins and in cores of protein fibrils. The antibodies have been found to lack reactivity with natively folded SOD1. In contrast, all react avidly with denatured/disordered SOD1, in which the corresponding peptide segments can adapt to the antigen-binding sites [14, 15]. In reaction with fibrils/aggregates, the binding of the anti-peptide antibodies is essentially “binary”. There is no response to the ordered core of protein aggregates/fibrils or to segments otherwise hidden. Non-recruited sequence elements, which have lost their native contacts and therefore are disordered, will react with the antibodies.

Usually lumbar, thoracic and cervical spinal cord, brainstem, brain (dissected free from midbrain, pons and medulla) and cerebellum were analyzed. The dissected tissues were homogenized with an Ultraturrax apparatus (IKA, Staufen, Germany) for 30 s and by sonication for 1 min in 25 volumes of ice-cold PBS containing an antiproteolytic cocktail (Complete, Roche Diagnostics, Basel, Switzerland). The tissue homogenates were added to 20 volumes of PBS containing 1% NP40, sonicated for 30 s, centrifuged at 1000×*g* for 10 min, and the supernatants collected for assay by binary epitope mapping.

Serial 1 + 1 dilutions of the supernatants were captured on cellulose acetate filters in a dot-blot apparatus. The filters were then cut in slices, incubated with the 15 antibodies and then developed similar to western immunoblots. Chemiluminescence of the blots was recorded in a ChemiDoc Touch Imaging System (BioRad) and analyzed with ImageLab software. To allow comparison and quantification, one homogenate of spinal cord from an end-stage *hSOD1*^*G93A*^ Tg mouse, kept frozen in aliquots, is designated as a standard (set to 1) and run in one or two lanes on all filters and stained with the 57–72 Ra-Ab. For an example, see Supplementary Fig. S1. All blots of all homogenates with all antibodies were quantified against this standard. To facilitate comparison of staining patterns, in some cases, the staining intensities of the 15 antibodies with individual homogenates were normalized against the staining of that homogenate with the 57–72 Ra-Ab (taken as 100%).

### Quantification of detergent-resistant hSOD1 aggregates

Spinal cord plus brain stem from end-stage mice were homogenized using an Ultraturrax followed by sonication in 25 volumes of PBS containing 1% NP40. Aliquots of the homogenates were further diluted 20-fold with NP40-containing PBS and again sonicated. The homogenates were made 3% in iohexol and added to ultracentrifugation tubes. PBS was then cautiously layered on top to fill the soft ultracentrifugation tubes. Following 2-h centrifugation at 175,000×*g*, the contents of hSOD1 in the pellets were determined with western blotting.

### Analysis of the total protein content of the seeds

Aliquots of the seed suspensions were mixed with equal volumes of 2 × SDS-PAGE sample buffer without glycerol, bromophenol blue and reductant and were boiled for 10 min. The protein contents were analyzed with the BCA protein assay (Pierce) using bovine serum albumin boiled in 1× sample buffer as standard.

### Immunoblots and quantifications

The western immunoblots were carried out as described [20] using antibodies raised in rabbits against peptides in the hSOD1 sequence as indicated in the figure legends. Chemiluminescence of the blots was recorded in a ChemiDoc Touch Imaging System and analyzed with ImageLab software (BioRad).

### Tissue handling and histopathology

The mice were killed by intraperitoneal injection of pentobarbital, decapitated and the spinal cords were removed by flushing with saline using a syringe inserted distally. The cords were then divided sagittally, and typically alternating right or left halves were immersion fixed for histopathology. Fixed spinal cords were divided into lumbar, thoracic and cervical segments, and each segment then cut into six parts which were processed as described below. The other half was likewise divided into lumbar, thoracic, and cervical segments and snap-frozen in − 80 °C for subsequent analysis of amounts and structures of hSOD1 aggregates. The brains were dissected and divided into right and left hemispheres, cerebellum and brainstem, and processed like the spinal cords. Fixed tissue was paraffin-embedded, and 4 µm thick sections were cut with a microtome (Microm HM400), and mounted on glass slides.

For immunohistochemistry, sections were immunostained according to the manufacturer’s recommendations using the Ventana Benchmark Ultra (Ventana Medical Systems Inc.). The sections were preincubated with Ventana Cell Conditioning Solution 2 (CC2) for 8 min followed by 4 min of Ventana Protease 1 solution. The primary 131–153 Mo-Ab was used at the concentration 1 µg/ml. Incubation time for primary antibody was 32 min. The bound primary antibodies were located with corresponding biotin-conjugated secondary antibodies coupled to a streptavidin–horseradish peroxidase conjugate and developed using 3,3′diaminobenzidine tetrahydrochloride as the precipitating enzyme product (brown color) (iView DAB detection kit, Ventana). Sections were counterstained with hematoxylin, washed, and mounted with Glycergel Mounting Medium (DakoCytomation). Micrographs were taken in a BX53 microscope (Olympus) with lamp set at fixed voltage, intensity adjusted by standard gray filters using a DP72 camera (Olympus). Images were acquired using the Olympus CellSens program (version 1.7) with standard settings and white balancing on out-of-sections areas.

For immunofluorescence, sections were double immunostained using 131–153 Ch-Ab for detecting SOD1 aggregates and GFAP (Z0334, DakoCytomation) for detecting astrocytes. As corresponding fluorescent-labeled secondary antibodies, Alexa flour A11039 and A21428 (Invitrogen) were used. The sections were examined by confocal laser microscopy using a Zeiss LSM 710 confocal microscope and were analyzed using the Zen 2011 SP7 software.

Twenty-one end-stage and six early-onset *hSOD1*^*G85R*^ Tg mice inoculated with the human hSOD1^G127X^–I seed and 18 *hSOD1*^*G85R*^ Tg mice inoculated with the human control seed were investigated. Furthermore, 13 non-inoculated 300-day-old and seven non-inoculated 200-day-old *hSOD1*^*G85R*^ Tg mice were also investigated. In each mouse, sections from lumbar, thoracic, and cervical spinal cord, brain stem, cerebellum and brain were examined.

Counting of motor neurons in the spinal cord ventral horns was performed on 20 end-stage *hSOD1*^*G85R*^ Tg mice inoculated with the human-derived hSOD1^G127X^ I and II seeds and 12 non-inoculated *hSOD1*^*G85R*^ Tg control mice (mean age 300 days). Formalin-fixed paraffin-embedded sections as described above were used. The sections were stained using an anti- ChAT antibody (Abcam; EPR16590, 1:750, 32 min incubation time) using the Ventana Benchmark Ultra (Ventana Medical Systems Inc.) automatic staining system and for preincubation we used the CC1 reagent (Ventana Medical Systems Inc.). All histological samples were whole-slide digitalized using a scanning system (Pannoramic 250, Plan-Apochromat, CIS_VCC_F52U25CL). Computer graphic analysis was performed with CaseViewer software, both from 3D Histotech Ltd., Budapest, Hungary. The ImageJ software (http://rsb.info.nih.gov/ij/docs/index.html) was used for morphological filtering. The ventral horn was defined as the area within the gray-matter border and anterior to a frontal plane through the central canal. Cell profiles immunopositive for ChAT were identified using the ImageJ software and profiles larger than 150 µm^2^ in this region were used in the analysis. As measurement of number in each level, the number of profiles for all sections analyzed in that segment in each animal (usually four sections in the thoracic and cervical segments and six in thoracic segments) was divided by the number of sections analyzed. Thus, in each mouse, 14 sections were normally analyzed.

### Statistics

Unless otherwise indicated in the text, two-sided Mann–Whitney *U* test was used for comparison of two groups and for three groups, Kruskal–Wallis test with Dunn's correction was used. A *p* ≤ 0.05 was considered significant.

## Results

### Human SOD1^G127X^ aggregates show strain A-like core structure

In the hSOD1^”G127X”^ mutation, an insertion of 4 nucleotides, tggg, causes an altered reading frame after codon G127 leading to the synthesis of a 5 aa long neosequence (GQRWK) followed by a premature stop codon [2]. To allow the assessment of whether template-directed aggregation occurred after the inoculations, the structure of hSOD1^G127X^ aggregates was determined. In the binary epitope-mapping assay, the binding of eight polyclonal anti-SOD1 peptide Ra-Abs to aggregates captured on a filter is probed [6]. The antibodies cover between them 90% of the sequence of the protein and only bind to disordered peptide segments [14, 15]. The ordered core of any fibrils is not seen, but non-recruited sequence elements that protrude in a disordered manner into the solvent will be stained. Since the amounts of hSOD1^G127X^ aggregates in the human ventral horns are minute [20, 21] and CNS from elderly humans produce strong obscuring backgrounds, no clear specific patterns appeared in the epitope-mapping assay. Therefore, we used spinal cords from end-stage *hSOD1*^*G127X*^ Tg mice, which contain about 30 times more hSOD1^G127X^ aggregates per unit wet weight, to profile the structure (Fig. 1b) [20, 21].

To enhance the resolution of the assay, the eight antibodies in the original protocol (blue columns) were complemented with seven Ra-Abs (red columns) raised against shorter hSOD1 peptides (Fig. 1). End-stage non-inoculated *hSOD1*^*G85R*^ Tg mice displayed typical strain A patterns: the N-terminal end recruited to the fibril core; the sequence element from ~ aa 45 to ~ aa 90 looping into the solvent; the ensuing segment to ~ aa 125 hidden/recruited; and the C-terminal end non-recruited and reactive with antibody. The patterns of aggregates from end-stage *hSOD1*^*G127X*^ Tg mice were comparable to those of the *hSOD1*^*G85R*^ Tg mice from the N-terminal to the 100–115 Ra-Ab. Unlike the case of hSOD1^G85R^ aggregates, however, there was a strong reaction with the 111–127 GQRWK Ra-Ab. The reactivities with the 115–121 and 123–127 GQRWK Ra-Abs suggest that this is principally due to the neopeptide in hSOD1^G127X^ aggregates being non-recruited and exposed. The patterns, thus, suggest that the fibril cores of hSOD1^G85R^ strain A aggregates and hSOD1^G127X^ aggregates could be equal.

### Inoculation of hSOD1^G127X^ aggregate seeds into spinal cord initiates premature fatal motor neuron disease

As recipients for the inoculations, we aimed at using Tg mice expressing full-length hSOD1s, since they are most representative for hSOD1-induced ALS in general, and also allow more complete aggregate structure profiling by binary epitope mapping. We chose the *hSOD1*^*G85R*^ Tg model since it displays a uniquely long time window before spontaneous hSOD1 aggregation and ensuing paretic disease develops (Supplementary Fig. S3). In the protocol used, 1-µl seed suspension is inoculated with stereotactic guidance into the left ventral horn of lumbar spinal cord of adult 100-day-old mice [7].

**Fig. 2:**
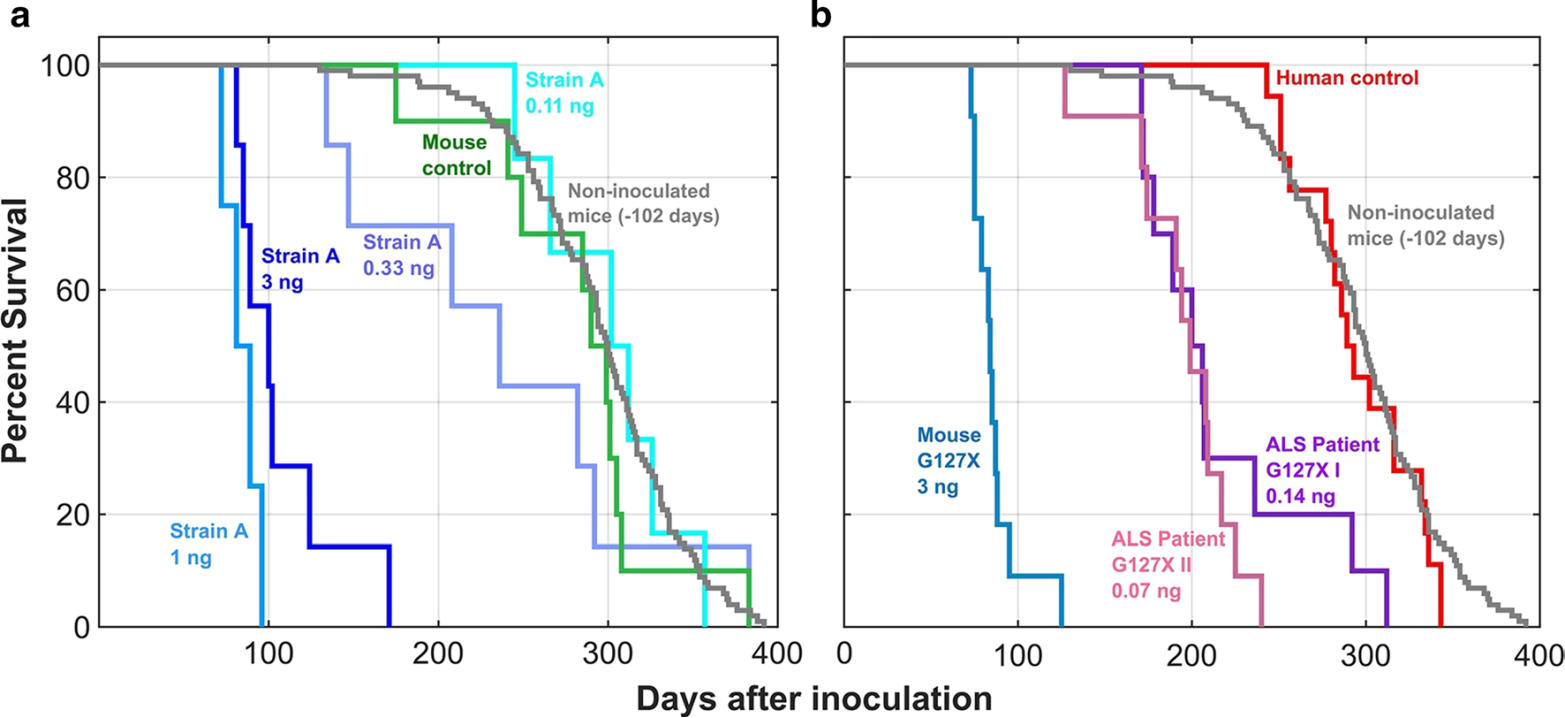
Inoculation of hSOD1 aggregate-containing seeds caused premature fatal motor neuron disease. **a** Inoculation of different dilutions of a hSOD1^G85R^ strain A seed compared with a non-transgenic control mouse seed. **b** Inoculation of a human and mouse-derived hSOD1^G127X^ seeds compared with a human control seed. As references both figures contain survival data for non-inoculated *hSOD1*^*G85R*^ Tg mice (*n* = 101) subtracted with the mean age (102 days) at which the inoculations took place. In **a** the post-inoculation survival lengths and significances for differences versus the mouse control were for the 3, 1, and 0.33 ng inoculations 107 ± 31 days, *p* < 0.0001; 85 ± 10 days, *p* < 0.002; and 301 ± 41, *p* < 0.14 (ns), respectively. The 0.33 ng data were significantly different from those of the non-inoculated mice (*p* < 0.04). For analysis of data in **b**. see Table 1

Whole spinal cords from end-stage *hSOD1*^*G127X*^ Tg mice and ventral horn specimen from a patient carrying the *hSOD1*^*G127X*^ mutation were used to prepare seeds. As controls, corresponding specimen from non-Tg C57BL/6 mice and a human dying from cardiovascular disease were used. Human hSOD1^G127X^ seeds were prepared with both the original (I) [7], and a subsequent somewhat modified protocol (II). The concentration of hSOD1^G127X^ aggregates in the murine seed, 3 ng/µl, was comparable to those in SOD1^G85R^ strain A and SOD1^D90A^ B seeds previously found to reproducibly transmit aggregation and fatal motor neuron disease [7]. The human seeds contained much less: 0.14 and 0.07 ng/µl (Supplementary Fig. S2). To explore the feasibility of the investigation, a dose–response study was carried out with a hSOD1^G85R^ strain A aggregate seed preparation which contained 3 ng/µl. Inoculations with 3 and 1 ng, respectively, resulted in essentially equal survival lengths, around 100 days after inoculation (Fig. 2a). The mice inoculated with 0.33 ng lived longer but still significantly shorter than non-inoculated *hSOD1*^*G85R*^ Tg mice. The difference versus those inoculated with the mouse control seed was, however, not significant. Further dilution resulted in survivals comparable to those of the control groups. This dose–response study suggested that there was a marginal chance to reveal any prion-like activity in the hSOD1^G127X^ aggregates in the human spinal cord-derived seeds.

Inoculation of the murine hSOD1^G127X^ seed caused a premature fatal motor neuron disease with a mean survival of 86 days, which is comparable to that of mice inoculated with the current and previous strain A hSOD1^G85R^ seeds (Fig. 2a, b, Table 1) [7]. Mice inoculated with the human-derived hSOD1^G127X^ I and II seeds lived longer after inoculation, 216 days and 196 days, respectively, but still significantly shorter than mice inoculated with either the human or murine control seeds (Fig. 2a, b, Table 1). The survivals of two of the mice extended well into the range of the control-inoculated mice, suggesting that the amounts of hSOD1^G127X^ aggregates in the seeds were close to the limit for reproducible initiation of motor neuron disease, as could be predicted from the dose–response study with the hSOD1^G85R^ strain A seed (Fig. 2a). The hSOD1^G127X^ aggregates in the human seed preparations seemed to be more potent than the strain A hSOD1^G85R^ aggregates in the murine seed.

**Fig. 3:**
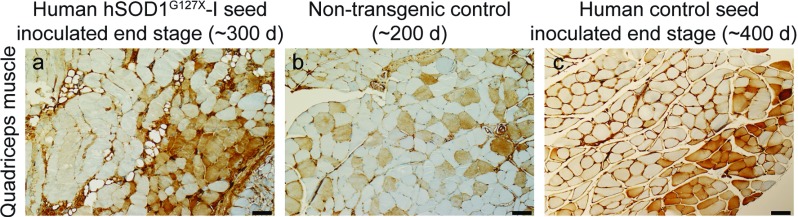
Fiber-type grouping in end-stage mice. **a**, **c** Micrographs of quadriceps muscle from end-stage *hSOD1*^*G85R*^ Tg mice inoculated with the human hSOD1^G127X^–I or control seeds and **b** a 200-day-old C57BL/6 control mouse. The muscles were stained with an antibody against slow myosin and the micrographs from the end-stage mice shows fiber type grouping indicating denervation-induced atrophy. Scale bars = 50 µm

Around the time of symptom onset, the mice began losing weight (Supplementary Fig. S4), which in the end stage was around 25% lower than the maxima (Table 1). There was fiber-type grouping in skeletal muscle in the inoculated Tg mice in the end stage (Fig. 3). Both these signs indicate denervation-induced muscle atrophy.

In end-stage human hSOD1^G127X^ seed-inoculated mice, the mean counts of ChAT-positive motor neurons per section were, compared with asymptomatic non-inoculated hSOD1^G85R^ Tg mice of the same age (~ 300 days), significantly lower in the lumbar, thoracic and cervical segments (Table 2). Furthermore, the profile areas (µm^2^) of the remaining motor neurons were also significantly smaller in all spinal cord segments (Table 2).

**Table 2:** Loss of motor neurons in inoculated mice

	Mean number of cells per section			Mean profile areas (µm^2^)		
	Human hSOD1^G127X^-seed inoculated mice	Non-inoculated SOD1^G85R^ Tg mice	*p* value	Human hSOD1^G127X^-seed inoculated mice	Non-inoculated SOD1^G85R^ Tg mice	*p* value
Cervical ventral horn	9.7 ± 4.6	13.0 ± 3.2	0.032	285 ± 72	422 ± 65	0.0001
Thoracic ventral horn	3.5 ± 1.6	5.2 ± 1.6	0.0063	270 ± 37	347 ± 67	0.003
Lumbar ventral horn	4.6 ± 2.7	6.2 ± 2.3	0.029	306 ± 89	437 ± 69	0.0003

The table shows data for mice investigated by immunohistochemistry with an antibody directed at ChAT. Data are shown as mean ± SD. The *p*-values denote the significance of differences between end-stage hSOD1^G127X^ seed-inoculated mice (*n* = 20) and aged-matched (~ 300 days) asymptomatic non-inoculated hSOD^G85R^ Tg mice (*n* = 12). The numbers given are the mean number of ChAT-positive profiles larger than 150 µm^2^ per section investigated. Usually four sections in the lumbar and cervical segments and six in the thoracic segment were investigated. The mean profile areas of remaining Chat-positive cells were significantly smaller in the inoculated end-stage mice

### Spread of templated hSOD1 aggregation along the neuraxis

**Fig. 4:**
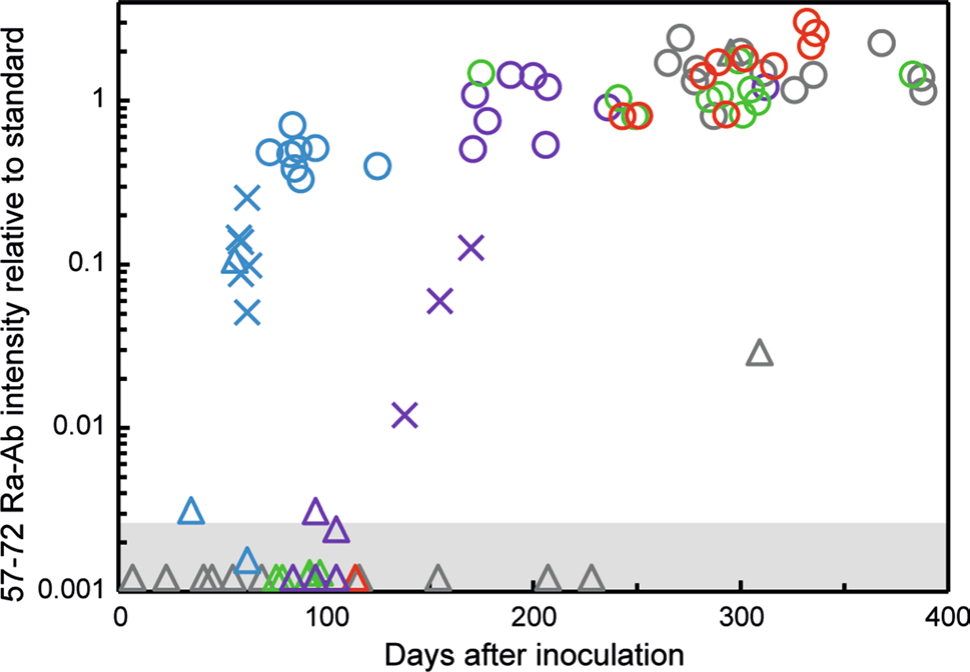
Content of strain A aggregates in whole spinal cords of inoculated and non-inoculated *hSOD1*^*G85R*^ Tg mice analyzed with binary epitope mapping. Triangles (triangle), crosses (cross) and circles (unfilled circle) indicate non-symptomatic, symptomatic and end-stage mice, respectively. Blue, violet, green, and red symbols indicate *hSOD1*^*G85R*^ Tg mice inoculated with the mouse hSOD1^G127X^ (blue unfilled triangle, blue unfilled cross, blue unfilled circle), human hSOD1^G127X^ I (purple unfilled triangle, purple unfilled cross, purple unfilled circle), mouse control (green unfilled triangle, green unfilled circle) and human control (red unfilled triangle, red unfilled circle) seeds, respectively. Gray indicates non-inoculated mice (gray unfilled triangle, gray unfilled circle) (lifespans minus 102 days, the mean age at which the inoculations took place). The shaded area indicates blank reactions

Spinal cords from end-stage *hSOD1*^*G85R*^ Tg mice inoculated with both the murine and human hSOD1^G127X^ seeds contained large amounts of aggregates as analyzed by both the epitope-mapping assay using the 57–72 ab (Fig. 4) and as detergent-resistant aggregates (Table 1). In the epitope-mapping assay, the aggregates displayed strain A patterns (Fig. 1c, d), comparable to those induced by the current (Fig. 1e) and previously reported hSOD1^G85R^ seeds [7]. The difference versus the pattern induced by a hSOD1^D90A^ strain B seed is great (Fig. 1f). We conclude that both the human and murine hSOD1^G127X^ seeds transmit strain A aggregation to the *hSOD1*^*G85R*^ Tg mice. Despite the differences between the aggregates formed by the truncated hSOD1^G127X^ and the full-length hSOD1^G85R^, the seemingly identical fibril cores appear to form the principal templates for aggregate growth (Fig. 1a, b). The mice in the various treatment groups reach the paralytic end stage within relatively narrow ranges of aggregation, but there is overall an increase in levels of end-stage aggregates, the older the mice are (Pearson correlation *R*^2^ = 0.419, *p* < 0.0001) (Table 1; Fig. 4). The reason is not clear, but could perhaps be caused by impaired clearance of cell debris and aggregates owing to microglial senescence [33].

**Fig. 5:**
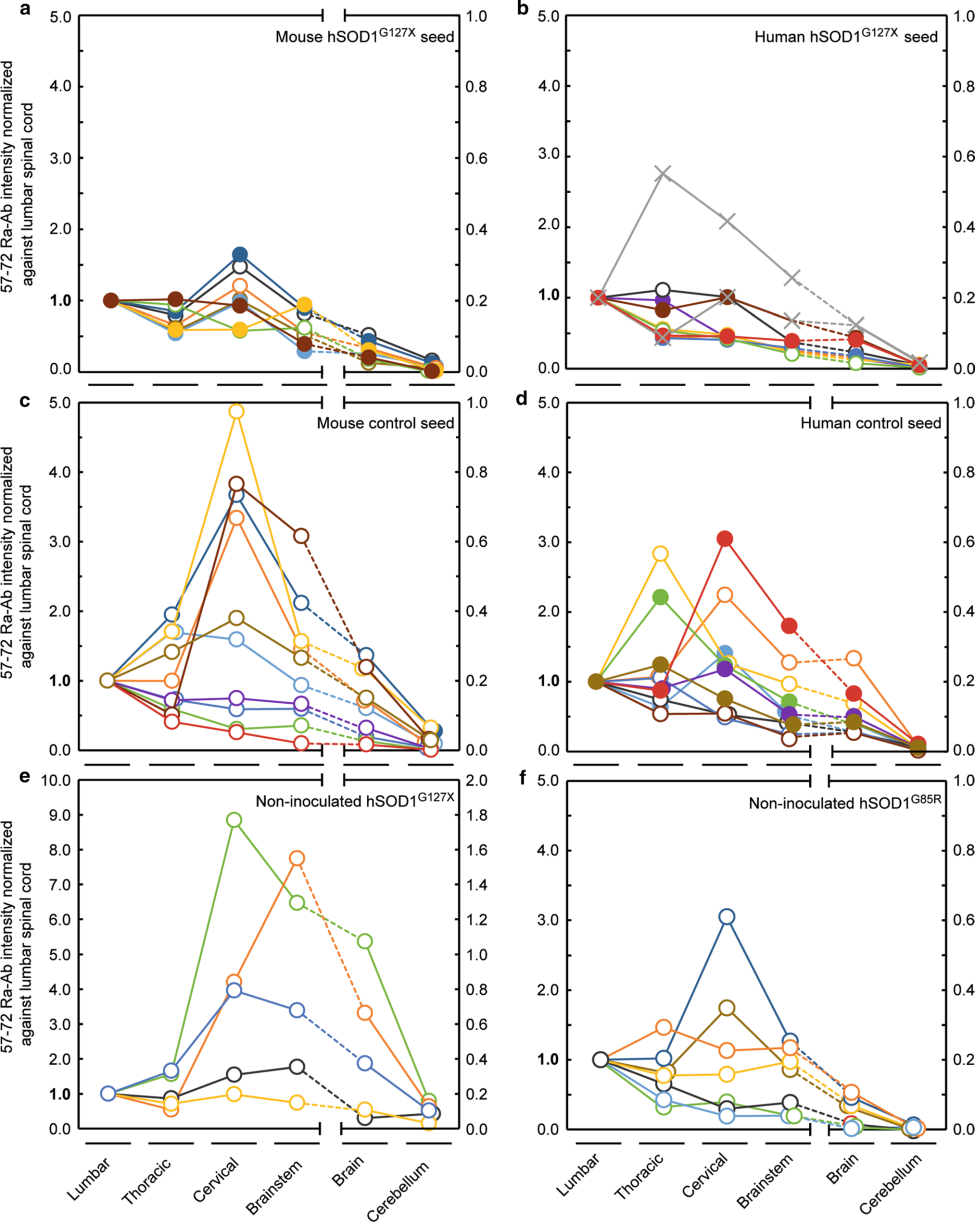
Distribution of strain A hSOD1 aggregation along the neuraxis in individual end-stage Tg mice. The aggregates were analyzed with binary epitope mapping using the 57–72 Ra-Ab. The levels in the segments were normalized against the levels in the lumbar spinal cord. Note the change in scale for brain and cerebellum. The results for individual mice are presented in different colors to improve distinction. **a**, **d** Results for *hSOD1*^*G85R*^ Tg mice inoculated with indicated seeds. In **a**, **b**, and **d** only the left (inoculation side, closed circles) or right halves (open circles) of the neuraxes were analyzed. In **b**, results for the two human hSOD1^G127X^-I seed-inoculated mice with lifespans equal to or longer than the mean survivals of the human control-inoculated and the non-inoculated mice are labeled (cross) (c.f. Fig. 2b). **e**, **f** Results for non-inoculated *hSOD1*^*G127X*^ and *hSOD1*^*G85R*^ Tg mice

The distribution of the strain A aggregation along the neuraxis was analyzed in end-stage mice. In the *hSOD1*^*G85R*^ Tg mice inoculated with the murine- and human-derived hSOD1^G127X^ seeds, the concentrations were essentially even between the spinal cord segments. The brain contained 10–20-fold less and aggregates were sparse in cerebellum (Fig. 5a, b). There were no significant differences between the left inoculation side (closed circles) and the right side (open circles) (Fig. 5a, b). This is comparable to the findings in the previous study, in which the side distributions were essentially equal in the paralytic end stage, whereas in the presymptomatic early stages, there were more aggregates in the left inoculation side [7]. The distributions were more variable between individual mice inoculated with the murine and human control seeds (Fig. 5c, d), and among *hSOD1*^*G127X*^ and *hSOD1*^*G85R*^ Tg mice spontaneously developing fatal motor neuron disease (Fig. 5e, f).

The sites of symptom onsets differed between the mice inoculated with hSOD1^G127X^ seeds and the control-inoculated and non-inoculated groups (*p* < 0.02 for all comparisons, two-sided Fisher´s exact test). All 17 *hSOD1*^*G85R*^ Tg mice inoculated with the murine hSOD1^G127X^ seed, and which were followed until onset or the end stage (c.f. Figs. 2, 4), showed paralysis onsets in the hind legs. Of 22 mice inoculated with the human hSOD1^G127X^ I and II seeds, 21 had distinct hind leg onsets and 1 combined hind leg/foreleg onset (the two mice with lifespans equal to or longer than the mean lifespans of the human control-inoculated or the non-inoculated mice were excluded from this analysis, c.f. Fig. 2c). Of the 28 human- and murine control-inoculated mice, 15 had hind leg onsets and 13 had combined hind leg and foreleg or foreleg onsets. Finally, among 13 monitored non-inoculated hSOD1^G85R^ mice, 8 had hind leg onsets and 5 had combined hind leg/foreleg or foreleg onsets.

### Histopathology

**Fig. 6:**
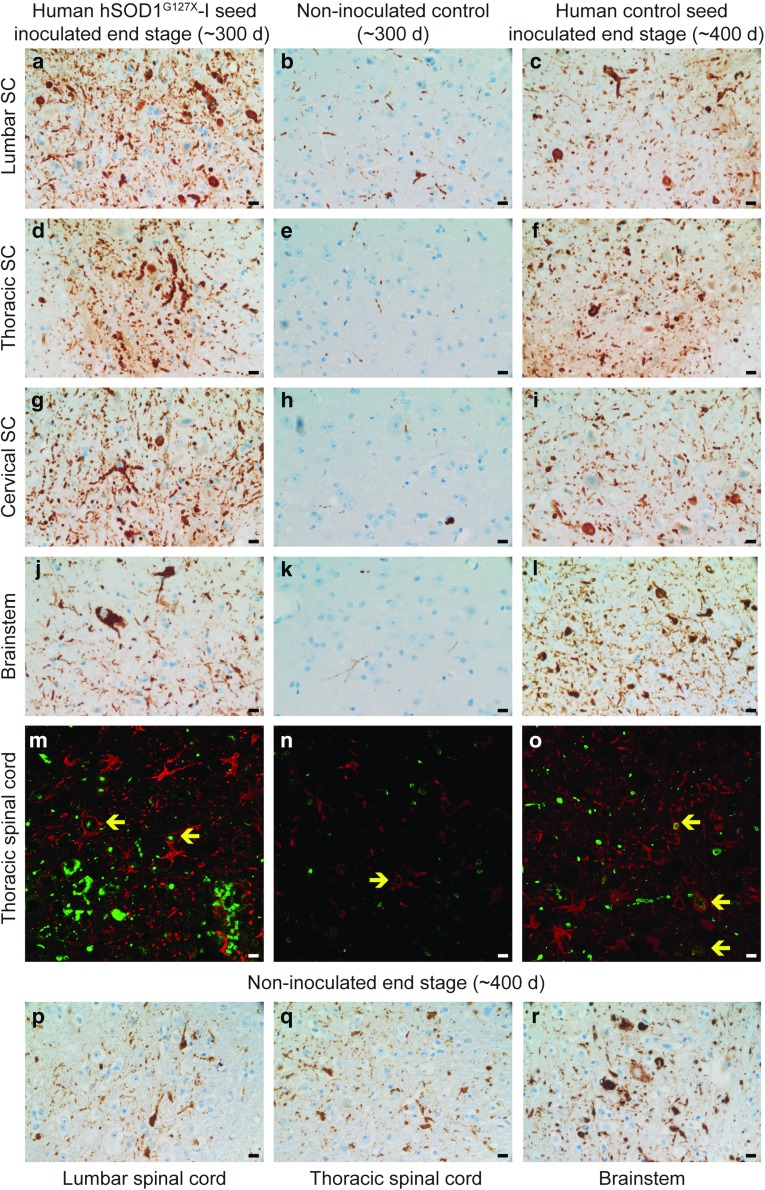
Histopathology of end-stage inoculated and asymptomatic non-inoculated *hSOD*^*G85R*^ Tg mice. **a–l** Sections were stained with the 131–153 Mo-Ab. For the non-inoculated 300-day-old control mice (middle column), a typical picture for the five mice showing sparse labeling is shown. In seven, no labeling was found and in one, the staining was comparable to that of end-stage mice. Note that while non-inoculated 300-day-old *hSOD1*^*G85R*^ Tg mice almost entirely show thread-like immunoreactivity (**b**, **e**, **h**, and **k**), the end-stage inoculated mice show widespread granular reactivity in motor neurons somas as well as neuropil threads (**a**, **c**, **d**, **f**, **g**, **i**, **j**, **l**). **m–o** Sections were stained with the 131–153 Ch-Ab (green) and an antibody against GFAP (red). Yellow arrows indicate colocalization of misfolded SOD1 and GFAP. Note the large difference between hSOD1^G127X^-I seed-inoculated (**m**) and age-matched control animals (**n**). **p**–**r** sections from end stage non-inoculated *hSOD1*^*G85R*^ Tg mice stained with the 131–153 Mo-Ab. Scale bars = 10 µm

Using the 131–153 Mo-Ab, no hSOD1 labeling was seen in ~ 200-day-old non-inoculated control *hSOD1*^*G85R*^ Tg mice. The findings varied in-between different ~ 300-day-old control mice with half of them (7/13) showing no labeling at all and 5 of the 13 showing only sparse labeling, mainly consisting of neuropil threads. Motor neuron somal staining was in these mice mainly seen at lumbar levels (Fig. 5b, e, h, k). One of the 13 mice, 309-day-old and showing early hind leg symptoms, displayed extensive staining in both neuronal somata and neuropil threads at all levels (data not shown) comparable to the findings in end-stage *hSOD1*^*G85R*^ Tg mice (e.g., Fig. 6f, i, l).

Albeit a small individual variation in-between different end-stage *hSOD1*^*G85R*^ Tg mice inoculated with the human hSOD1^G127X^seeds (~ 300-day old) were seen, no consistent differences existed between hSOD1^G127X^–I or hSOD1^G127X^–II seeds when analyzed using the 131–153 Mo-Ab. At all levels, there was extensive occurrence of neuropil threads and small granular somal inclusions (Fig. 6a, d, g, j). Noteworthy is that not all motor neurons showed inclusions. There were fewer threads in the brain stem, where the highest numbers were seen in the dorsal part of the medulla oblongata (Fig. 6J). No somatic granular inclusions were seen in the cortex, and only in one of eight investigated animals were neuropil threads seen there (not shown).

The findings both with regard to neuropil threads and soma inclusions as seen with the 131–153 Mo-Ab in end-stage *hSOD*^*G85R*^ Tg mice inoculated with the human control seed preparation (~ 400-day-old) (Fig. 6c, f, i, l) were comparable to those in the non-inoculated end-stage *hSOD1*^*G85R*^ Tg mice (~ 400-day-old) (Fig. 6p–r) and marginally less than seen in the human hSOD1^G127X^-seed inoculated mice. Thus, the human control seed or the surgical trauma do not seem to have any effect on end-stage aggregate accumulation.

The amount of threads in the spinal cord of the end-stage human hSOD1^G127X^-1 seed-inoculated mice (~ 300-day old) (Fig. 6a, d, g) exceeded that of the 100-day-older end-stage human control-inoculated *hSOD1*^*G85R*^ Tg mice (Fig. 6c, f, i), while the amount of threads in the brainstem was higher in the latter (Fig. 6l). Somas with small granular inclusions were generally more numerous in the end-stage human control-inoculated *hSOD1*^*G85R*^ Tg mice (Fig. 6l). The occurrence of threads in the human hSOD1^G127X^-1 seed-inoculated animals seems to be a late phenomenon, since in onset and pre-onset such mice threads were hardly seen at all (not shown).

It has previously been reported that hSOD1 inclusions are abundant in astrocytes in the *hSOD1*^*G85R*^ Tg model [8]. To examine astrocyte aggregate pathology in the current study, we utilized double-labeling confocal laser immunohistochemistry using the 131–153 Ch-Ab and an antibody directed against the astrocytic marker GFAP (Fig. 6m–o). We found that only few of the SOD1 inclusions in end-stage *hSOD1*^*G85R*^ Tg mice inoculated with the human hSOD1^G127X^-1 seed (~ 300-day-old) were associated with astrocytes (Fig. 6m), with the major proportion either freely located in the neuropil or within motor neurons. In age-matched non-inoculated hSOD1^G85R^ Tg mice (~ 300-day old), almost none of the few threads and granular small inclusions immunopositive for SOD1 were associated with astrocytes (Fig. 6n). Finally, in end-stage human control-inoculated *hSOD1*^*G85R*^ Tg mice (~ 400-day-old), the degree of association of granular hSOD1 inclusions with astrocytes appeared to be comparable to that in end-stage hSOD1^G127X^-1 seed-inoculated mice (~ 300-day-old) (Fig. 6o). Overall, the astrocyte hSOD1 pathology appeared to be less advanced in our current *hSOD1*^*G85R*^ Tg strain than in the original strain [8].

## Discussion

The present study provides evidence for the presence of hSOD1 aggregates with prion-like properties in the spinal ventral horn from a patient carrying the *hSOD1*^*G127X*^ mutation. The two different patient-derived aggregate seed preparations both transmitted a premature fatal motor neuron disease with hallmarks of human ALS: focal onset of progressive paralysis, loss of motor neurons and wasting. Concomitantly aggregation of hSOD1^G85R^ developed in the inoculated mice, which spread all along the neuraxis. The aggregates in end-stage *hSOD1*^*G127X*^ Tg mice had a strain A-like core structure and the induced aggregates displayed strain A patterns (Fig. 1c–e) [7]. This suggests that the aggregation transmitted by the murine SOD1^G127X^ seed was templated. The human hSOD1^G127X^ seed also induced strain A hSOD1^G85R^ aggregation, but since the inducing aggregates could not be profiled, we cannot draw any firm conclusions regarding templating. Of note, induced aggregation in *hSOD1*^*G85R*^ Tg mice seems to replicate the structure of the inducing species: strain B aggregates from *hSOD1*^*D90A*^ Tg mice induce formation of strain B aggregates which do not arise spontaneously in *hSOD1*^*G85R*^ Tg mice (Fig. 1f) [6, 7]. Further studies are needed to address the mechanisms by which the hSOD1 aggregation spreads in the CNS and causes the fatal neurotoxicity.

Aggregates of disease-relevant proteins have shown seeding effects in other models of neurodegenerative disease [11, 12, 26, 28, 29]. The relevance of the findings for the diseases in humans has, however, been questioned [13, 36]. In some cases, the induced pathology poorly mimics the modeled human disease, and the amounts of aggregates in the seeds and levels of expression of the disease-relevant proteins in the animals are considered unrealistically high. It is also suggested that the protein aggregation is related to specific vulnerabilities of subsets of neurons and might mainly occur cell autonomously in these.

In the current study, most of these concerns have been addressed. (1) As in humans, the fatal disease in *hSOD1*^*G85R*^ Tg mice has a middle-age onset, and demonstrates all the major hallmarks of human ALS. (2) The concentration of the hSOD1^G85R^ protein in the recipient mice is no higher than those of the endogenous murine SOD1 or hSOD1 in the human CNS [8, 21]—although the proportion that is disordered is higher [38, 39]. (3) The amounts of SOD1^G127X^ aggregates in the 1-µl inoculates were minute: 0.14–0.07 ng. This is around 500 times less than the amount of murine or human SOD1 in the same volume of spinal cord tissue from controls [17, 22], and ~ 30 times less than the amount of aggregated hSOD1^G127X^ present in 1-µl (mg) ventral horn from patients carrying the *hSOD1*^*G127X*^ mutation [19, 20]. Finally, the amounts of hSOD1^G85R^ aggregates in end-stage mice were > 30,000 times greater than the amounts of hSOD1^G127X^ aggregates that were inoculated. Thus, the seeding efficiency of the patient-derived hSOD1^G127X^ aggregates was extremely high, and they transmitted disease to the Tg mice at levels that were much lower than those found in motor areas of hSOD1^G127X^ patients. (4) The nature of the selective vulnerability of motor areas to SOD1-induced toxicity is still poorly understood. We have previously reported on two properties that would enhance the vulnerability to aggregation. Spinal cords from Tg model mice show a marked enrichment of disordered hSOD1 monomers [38, 39], which are the necessary substrates for nucleation and growth of aggregate fibrils [10, 16, 25]. Autophagy is found to be important for retarding hSOD1 aggregation in the transgenic models, and spinal motor areas in humans have apparently a low inherent such capacity [35]. These two properties could conceivably enhance autonomous hSOD1 aggregation in cells compromised by other neurotoxic mechanisms, but would certainly also increase the risk of stochastic initiation and subsequent prion-like spread of hSOD1 aggregation preferentially in the motor areas. The patterns of spontaneous hSOD1 aggregation in the murine models support the latter possibility. Here, and previously, we have shown that strain A and B hSOD1 aggregate seeds initiate templated hSOD1 aggregations which spread from the site of inoculation, from which area also the initial motor symptoms derive [7]. In *hSOD1*^*G85R*^ Tg mice that spontaneously develop disease or are inoculated with human or murine control seeds, the aggregation seems to initiate and spread from random positions along the spinal cord, with symptom onsets related to the segments with highest aggregate levels in the individual mice [7].

We conclude that hSOD1 aggregate seeds prepared from spinal ventral horn from a patient carrying a *hSOD1* mutation when inoculated in hSOD1-expressing mice initiated spreading hSOD1 aggregation concomitantly with fatal motor neuron disease. The potency of the seeds was extremely high, and disease was initiated in the Tg mice by levels of hSOD1^G127X^ aggregates much lower than those found in the human motor system. Our results suggest that prion-like spread of hSOD1 aggregation could be the primary pathogenic mechanism, not only in *hSOD1* Tg models, but also in hSOD1-induced ALS in humans.

## Electronic supplementary material

Below is the link to the electronic supplementary material.

Supplementary material 1 (PDF 1044 kb)
