# Supplementary material for: Mutant superoxide dismutase aggregates from human spinal cord transmit amyotrophic lateral sclerosis
Source: Acta Neuropathol. 2018 Oct 3;136(6):939–53. doi: 10.1007/s00401-018-1915-y (PMC6280858; doi:10.1007/s00401-018-1915-y)
Supplement: Supplementary file 1 — Supplementary material 1 (PDF 1044 kb) [file 401_2018_1915_MOESM1_ESM.pdf]

Supplementary figures

Mutant superoxide dismutase aggregates from human spinal cord transmit amyotrophic lateral sclerosis

Elaheh Ekhtiari Bidhendi et al.

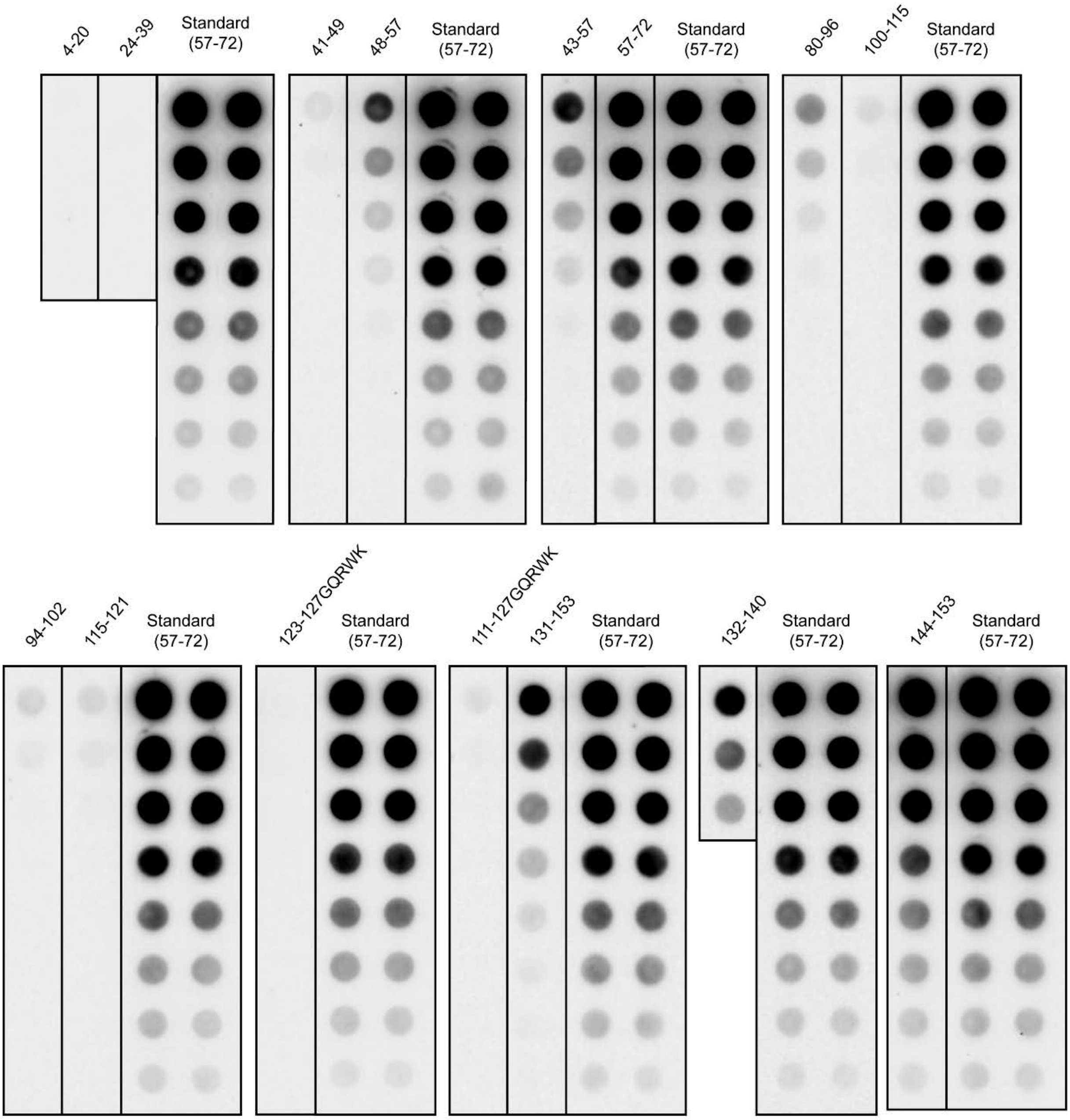

Supplementary Fig. S1. Binary epitope mapping of hSOD1^{G85R} aggregates in homogenate of spinal cord from an end-stage non-inoculated hSOD1^{G85R} Tg mouse. This mapping is one of those that compose Fig. 1a. Serial 1+1 dilutions of the 1/520 homogenate was applied to a cellulose acetate filter in a dot-blot apparatus. The filter was then sliced and the lanes stained with one or two (different lanes) of the antipeptide antibodies as indicated in the figure (c.f. Materials and methods). A 1/520 homogenate of spinal cord from an end-stage hSOD1^{G93A} Tg mouse is designated as a standad (set to 1), and was applied to two of the 12 lanes on each filter and was stained in parallel with the 57-72 Ra-Ab. In the figure these standard blots are shown together with the test blots on the individual filters. All blots of all homogenates with all antibodies are in this manner quantified against the standard.

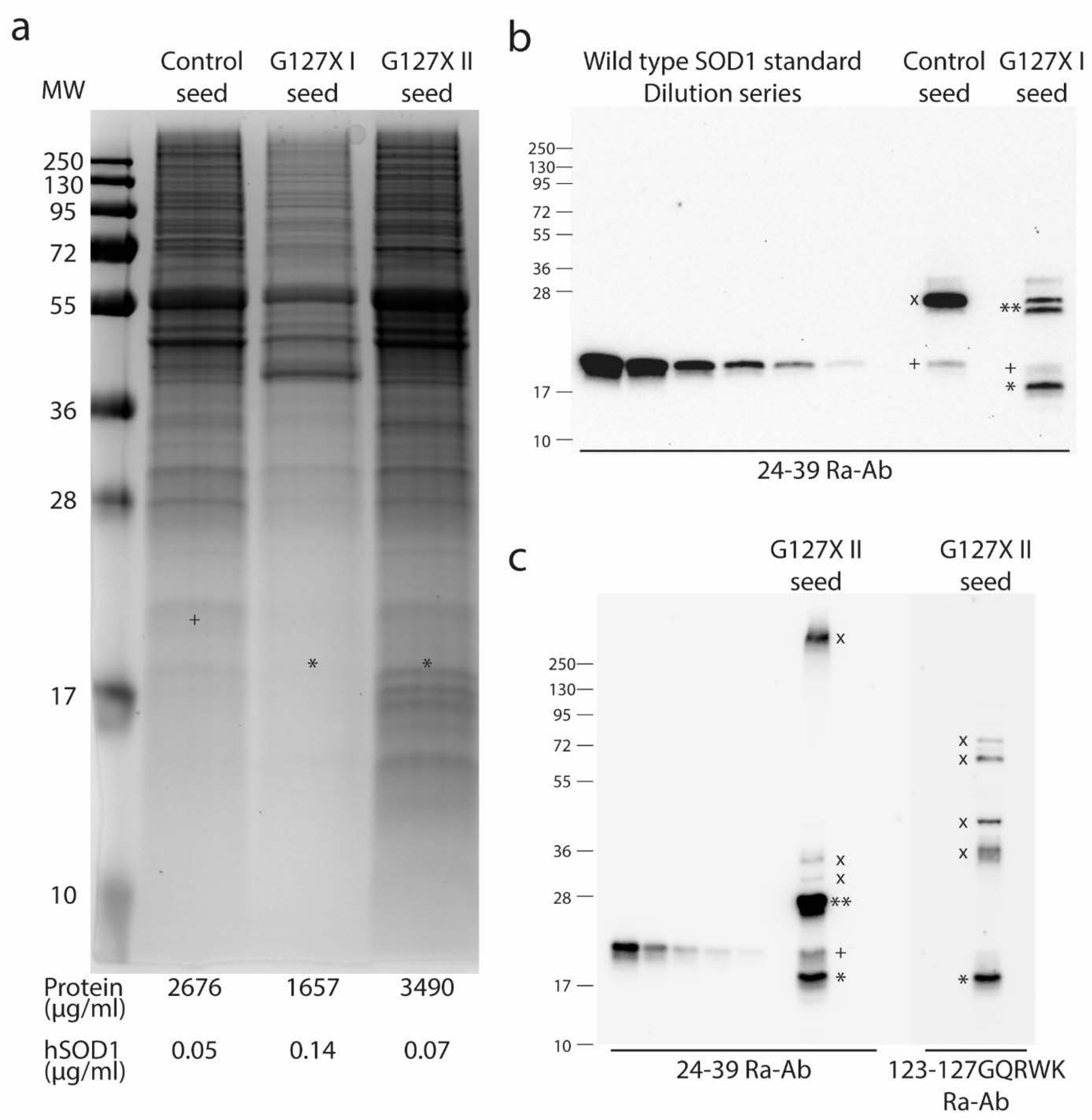

Supplementary Fig. S2 Characterization of the human hSOD1^{G127X} and human control seeds. The symbols indicate the following: ∗, hSOD1^{G127X}; ∗∗, hSOD1^{G127X} homodimer; +, wild type hSOD1; ×, unidentified cross-reacting proteins. **a** SDS-PAGE gel stained with Coomassie Brilliant Blue. The positions of wild type hSOD1 and hSOD1^{G127X} were deduced by comparison with positions of the hSOD1 variants in western blots in **b** and **c** relative to molecular weight markers. **b** Western blot of a dilution series of a human hSOD1 standard, the human control seed (prepared with protocol I), and the hSOD1^{G127X} I seed stained with the 24-39 Ra-Ab. **c** Western blot of a dilution series of a wild type hSOD1 standard and the hSOD1^{G127X} II seed stained with the 24-39 Ra-Ab, and the hSOD1^{G127X} II seed stained with the mutant-specific 123-127GQRWK Ra-Ab. The samples were run on the same SDS-PAGE and blotted onto the same membrane.

The hSOD1^{G127X} homodimer is seen in all western blots of extracts of CNS tissues from human mutation carriers and hSOD1^{G127X} Tg mice using the 24-39 Ra-Ab [1, 8]. It is covalently coupled, and

in western blots with this antibody it becomes about 10-fold overestimated relative to the monomer [1]. The band is therefore not added to the quantification of the mutant SOD1. The homodimer is not stained by the 123-127GQRWK antibody, perhaps suggesting that the covalent attachment occurs near the C-terminal end of the mutant SOD1 subunits [7]. The ×-marked bands in **b** and **c** are seen with only one of the two antibodies suggesting they do not represent hSOD1^{G127X} and are likely cross-reacting proteins, which have not been identified. Note that small amounts of wild-type hSOD1 are seen in the hSOD1^{G127X} seeds and the human control seed.

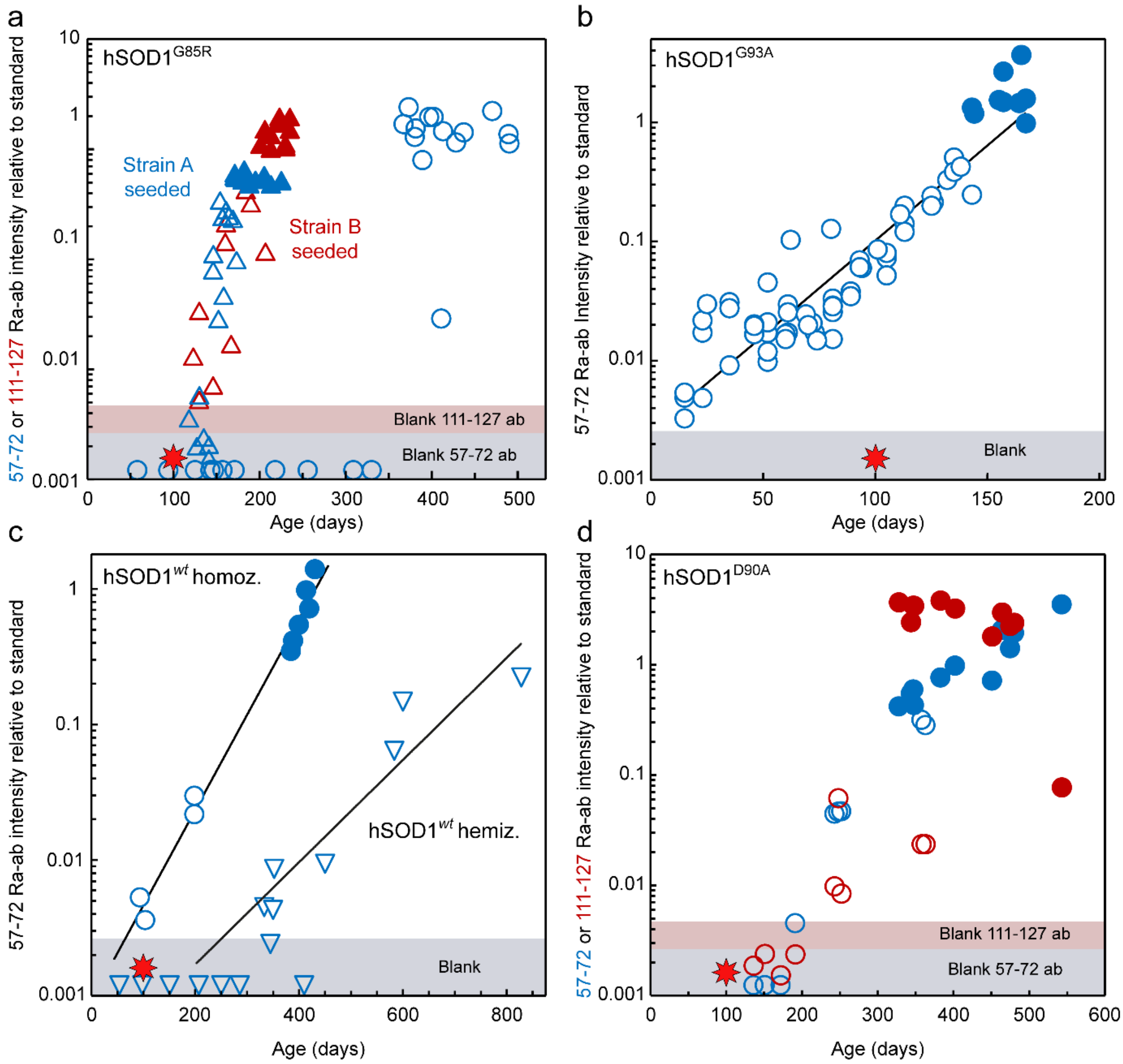

Supplementary Fig. S3 Spontaneous aggregation in murine Tg ALS models and choice of model for seeding. Since murine and human SOD1s do not coaggregate, seed inoculations into non-transgenic mice are expected not to result in transmission of aggregation [2-4, 10]. Still, we inoculated a hSOD1^{G85R}–derived strain A seed into ten non-transgenic C57Bl/6 mice. Three were sacrificed owing to wounds (after 31, 427 and 533 d), and two died after 176 and 278 d for unknown reasons. The remaining five mice lived until they were 633, 699, 721, 735, and 792 days old, and died owing to old age and/or tumors. None of the ten mice showed paretic symptoms or SOD1 aggregation [3].

Aggregation in spinal cords of the five examined hSOD1 Tg ALS models was determined with binary epitope mapping [2]. In the choice, we assume that the rate of seeded aggregation would be comparable to the rate of spontaneously evolving aggregation following initiation. Filled symbols indicate end-stage paralytic mice. (✱), position of spinal cord containing 5 ng hSOD1 aggregates, the largest amount that can be seeded using our protocols. a The long time window before significant

strain A aggregation appears in the hSOD1^{G85R} Tg model (○, ●) is unique. Strain A (△, ▲, 57-72 Ra-Ab) and strain B (△, ▲, 111-127GQRWK Ra-Ab) seeded aggregations are easily distinguished [3]. **a** and **c** In hSOD1^{G93A} [6] and homozygous hSOD1^{WT} [5] mice (○, ●) spontaneous strain A aggregations are at 100 d too advanced for seeding. The slow rate of strain A aggregation in hemizygous hSOD1^{WT} Tg mice [6] (▽) suggests that any successful seeding would reach paralysis-causing concentrations of aggregates at around 800 d, when mice already are senescent. **d** Both strain A (○, ●, 57-72 Ra-Ab) and strain B (○, ●, 111-127GQRWK Ra-Ab) aggregations appear in all hSOD1^{D90A} Tg mice [2, 9], making this model too complex for general seeding purposes.

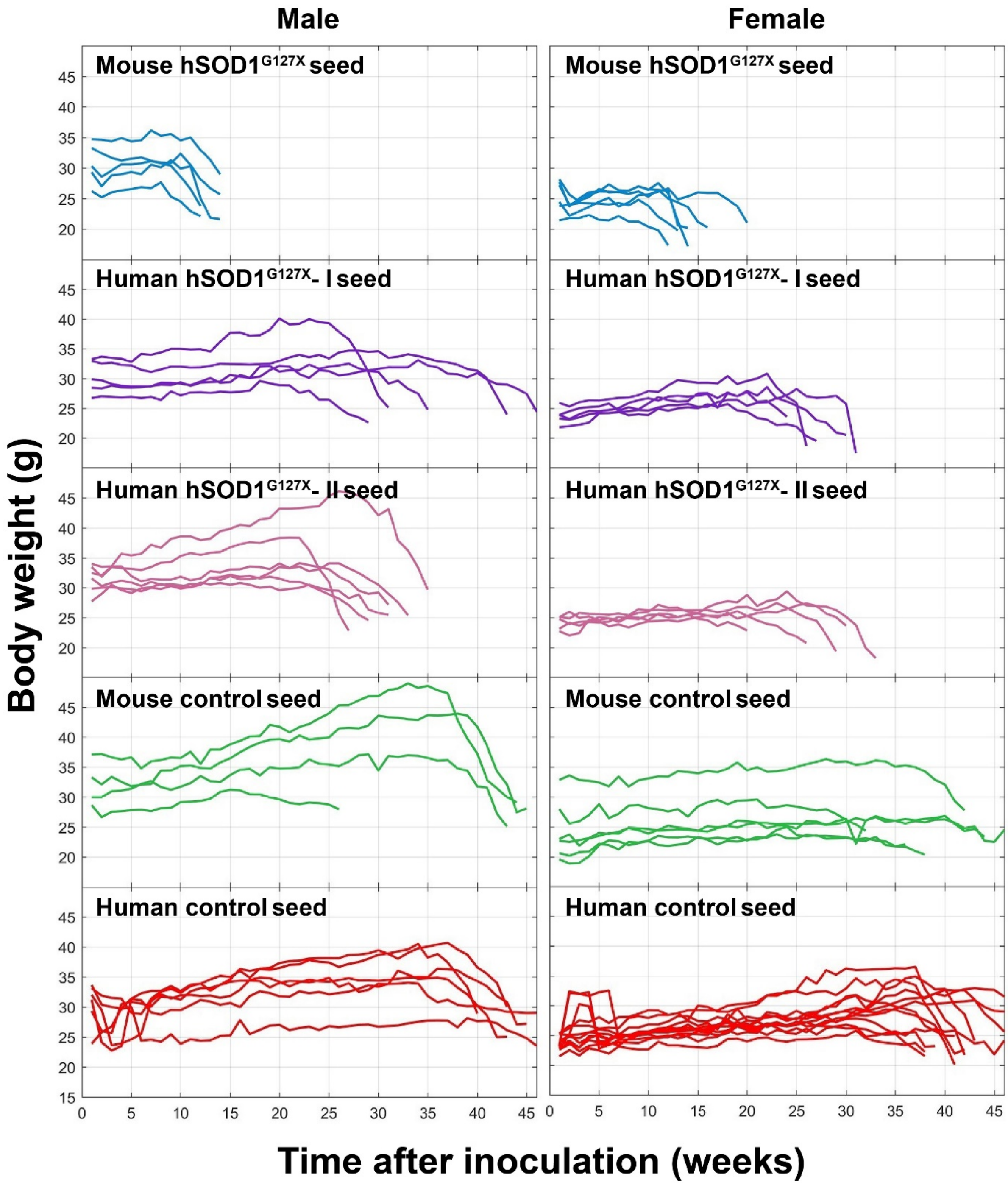

Supplementary Fig. S4 Weight loss in inoculated *hSOD*^{G85R} Tg mice. Weight developments of *hSOD1*^{G85R} Tg mice inoculated with indicated seeds are shown. The mice were weighed once a week. At around the onset of symptoms all mice began losing weight. For calculations on weight losses, see Table 1.

References

1 Bergemalm D, Forsberg K, Jonsson PA, Graffmo KS, Brannstrom T, Andersen PM, Antti H, Marklund SL (2009) Changes in the spinal cord proteome of an amyotrophic lateral sclerosis murine model determined by differential in-gel electrophoresis. Mol Cell Proteomics 8: 1306-1317 Doi 10.1074/mcp.M900046-MCP200

2 Bergh J, Zetterstrom P, Andersen PM, Brannstrom T, Graffmo KS, Jonsson PA, Lang L, Danielsson J, Oliveberg M, Marklund SL (2015) Structural and kinetic analysis of protein-aggregate strains in vivo using binary epitope mapping. Proc Natl Acad Sci U S A 112: 4489-4494 Doi 10.1073/pnas.1419228112

3 Bidhendi EE, Bergh J, Zetterstrom P, Andersen PM, Marklund SL, Brannstrom T (2016) Two superoxide dismutase prion strains transmit amyotrophic lateral sclerosis-like disease. J Clin Invest 126: 2249-2253 Doi 10.1172/JCI84360

4 Grad LI, Guest WC, Yanai A, Pokrishevsky E, O'Neill MA, Gibbs E, Semenchenko V, Yousefi M, Wishart DS, Plotkin SSet al (2011) Intermolecular transmission of superoxide dismutase 1 misfolding in living cells. Proc Natl Acad Sci U S A 108: 16398-16403 Doi 10.1073/pnas.1102645108

5 Graffmo KS, Forsberg K, Bergh J, Birve A, Zetterstrom P, Andersen PM, Marklund SL, Brannstrom T (2013) Expression of wild-type human superoxide dismutase-1 in mice causes amyotrophic lateral sclerosis. Hum Mol Genet 22: 51-60 Doi 10.1093/hmg/dds399

6 Gurney ME (1994) Transgenic-mouse model of amyotrophic lateral sclerosis. N Engl J Med 331: 1721-1722

7 Jonsson PA, Bergemalm D, Andersen PM, Gredal O, Brannstrom T, Marklund SL (2008) Inclusions of amyotrophic lateral sclerosis-linked superoxide dismutase in ventral horns, liver, and kidney. Ann Neurol 63: 671-675 Doi 10.1002/ana.21356

8 Jonsson PA, Ernhill K, Andersen PM, Bergemalm D, Brannstrom T, Gredal O, Nilsson P, Marklund SL (2004) Minute quantities of misfolded mutant superoxide dismutase-1 cause amyotrophic lateral sclerosis. Brain 127: 73-88 Doi 10.1093/brain/awh005 awh005 [pii]

9 Jonsson PA, Graffmo KS, Brannstrom T, Nilsson P, Andersen PM, Marklund SL (2006) Motor neuron disease in mice expressing the wild type-like D90A mutant superoxide dismutase-1. J Neuropathol Exp Neurol 65: 1126-1136 Doi 10.1097/01.jnen.0000248545.36046.3c 00005072-200612000-00004 [pii]

10 Qualls DA, Prudencio M, Roberts BL, Crosby K, Brown H, Borchelt DR (2013) Features of wild-type human SOD1 limit interactions with misfolded aggregates of mouse G86R Sod1. Mol Neurodegener 8: 46 Doi 10.1186/1750-1326-8-46
